# A comparison of random-field-theory and false-discovery-rate inference results in the analysis of registered one-dimensional biomechanical datasets

**DOI:** 10.7717/peerj.8189

**Published:** 2019-12-10

**Authors:** Hanaa Naouma, Todd C. Pataky

**Affiliations:** 1 Bioengineering Course/Graduate School of Science and Technology, Shinshu University, Ueda, Nagano, Japan; 2 Department of Human Health Sciences/Graduate School of Medicine, Kyoto University, Kyoto, Japan

**Keywords:** Time series analysis, Random field theory, False discovery rate, Type I error rate, Dynamics, Kinematics, Forces, Biological systems, Biomechanics

## Abstract

**Background:**

The inflation of falsely rejected hypotheses associated with multiple hypothesis testing is seen as a threat to the knowledge base in the scientific literature. One of the most recently developed statistical constructs to deal with this problem is the false discovery rate (FDR), which aims to control the proportion of the falsely rejected null hypotheses among those that are rejected. FDR has been applied to a variety of problems, especially for the analysis of 3-D brain images in the field of Neuroimaging, where the predominant form of statistical inference involves the more conventional control of false positives, through Gaussian random field theory (RFT). In this study we considered FDR and RFT as alternative methods for handling multiple testing in the analysis of 1-D continuum data. The field of biomechanics has recently adopted RFT, but to our knowledge FDR has not previously been used to analyze 1-D biomechanical data, nor has there been a consideration of how FDR vs. RFT can affect biomechanical interpretations.

**Methods:**

We reanalyzed a variety of publicly available experimental datasets to understand the characteristics which contribute to the convergence and divergence of RFT and FDR results. We also ran a variety of numerical simulations involving smooth, random Gaussian 1-D data, with and without true signal, to provide complementary explanations for the experimental results.

**Results:**

Our results suggest that RFT and FDR thresholds (the critical test statistic value used to judge statistical significance) were qualitatively identical for many experimental datasets, but were highly dissimilar for others, involving non-trivial changes in data interpretation. Simulation results clarified that RFT and FDR thresholds converge as the true signal weakens and diverge when the signal is broad in terms of the proportion of the continuum size it occupies. Results also showed that, while sample size affected the relation between RFT and FDR results for small sample sizes (<15), this relation was stable for larger sample sizes, wherein only the nature of the true signal was important.

**Discussion:**

RFT and FDR thresholds are both computationally efficient because both are parametric, but only FDR has the ability to adapt to the signal features of particular datasets, wherein the threshold lowers with signal strength for a gain in sensitivity. Additional advantages and limitations of these two techniques as discussed further. This article is accompanied by freely available software for implementing FDR analyses involving 1-D data and scripts to replicate our results.

## Introduction

Multiple testing refers to performing many tests on the same dataset. This scenario is common in experimental research fields such as bioinformatics (Fernald et al., 2011), Molecular biology (Pollard, Pollard & Pollard, 2019), and medicine (Banerjee, Jadhav & Bhawalkar, 2009) which consider multiple dependent variables when drawing statistical conclusions. Usually an acceptable cutoff probability *α* of 0.05 or 0.01 (Type I error rates) is used for decision making. However, with the growing number of hypotheses being simultaneously tested, the probability of falsely rejecting hypotheses has become high (James Hung & Wang, 2010). In biomechanics, multiple testing problems are one of the major causes of a “confidence crisis of results” emerging in the field (Knudson, 2017), with 73% to 81% of applied biomechanics original research reports employing uncorrected multiple statistical analyses (Knudson, 2009). There is therefore an urgent need to both adopt multiple testing procedures and consider the differences amongst them.

The simplest method for handling multiple testing is the Bonferroni adjustment. However, this adjustment assumes independence (i.e., zero correlation) amongst the multiple tests, so is an extreme way to control false positives which can increase the likelihood of false negatives, especially amongst non-independent tests (Nichols & Hayasaka, 2003; Abdi, 2007; Pataky, Vanrenterghem & Robinson, 2015). In neuroimaging, for example, Bonferroni adjustments fail to consider correlation due to spatiotemporal data smoothness. Thus, there is a need for an alternative multiple testing procedure to restore the balance between false positives and false negatives.

Biomechanics is a scientific field which uses mechanical principles to understand the dynamics of biological systems. Measurements of motion and the forces underlying that motion are often analyzed as temporal one-dimensional (1-D) continua. Prior to analysis, these data are often registered to a common temporal domain, resulting in homologous data representation over a 1-D domain of 0%–100% (Sadeghi et al., 2003). 1-D biomechanical datasets like these are used in a large variety of studies. For example: to assess wearable technology effects on spine movement (Papi, Koh & McGregor, 2017), to understand arm swing contributions to vertical jump dynamics (Lees, Vanrenterghem & Clercq, 2004) and to study tendon-to-bone healing in dogs (Rodeo et al., 1993).

In biomechanics literature, the most common analysis method is to extract zero-dimensional (0-D) metrics such as local extrema (Pataky, Vanrenterghem & Robinson, 2015), integrals or means from 1-D measurements. Reducing 1-D data, which often represents complex temporal dynamics, to a single discrete number is non-ideal, not only because it ignores many aspects of the 1-D data, but also because this approach is often inconsistent with the experiment’s null hypothesis, which usually pertains to kinematics or dynamics in general, and not specifically to the extracted 0-D metrics. For instance, gait researchers who collect knee flexion/extension data often record this variable over time (e.g., 0–100% gait cycle), but rarely make hypotheses regarding the specific times at which scientifically relevant signals are expected to occur, or specific time series features like range of motion (Pataky, Robinson & Vanrenterghem, 2013). They instead extract 0-D scalars like maximum flexion angle, often because, when the data are visualized, these features appear to embody the instants of maximum effect size (Morgan & O’Connor, 2019; Le Sant et al., 2019). This scalar extraction approach not only fails to consider the whole movement, but also increases the probability of creating/ eliminating statistical significance. This approach has been termed “regional focus bias”, or *ad hoc* feature selection, and it can greatly increase the risk of incorrectly rejecting the null hypothesis (Pataky, Robinson & Vanrenterghem, 2013).

An alternative to 0-D metrics extraction, whole-trajectory 1-D analyses, emerged in the Biomechanics literature over the last two decades. The main 1-D techniques include: functional data analysis (FDA) (Ramsay & Silverman, 2005), principle component analysis (PCA) (Daffertshofer et al., 2004) and statistical parametric mapping (SPM) (Pataky, Robinson & Vanrenterghem, 2013). PCA is a dimensionality reduction technique and does not provide a method for hypothesis testing, so cannot easily be compared to the other two methods. FDA encompasses a variety of inferential procedures used to analyze 1-D data, including nonparametric permutation methods (Ramsay & Silverman, 2005; Warmenhoven et al., 2018). Since there are many existing FDA procedures of varying complexity, in this study we consider only SPM, which is simpler than FDA because it utilizes a relatively simple random field theory (RFT) inferential procedure, which requires just two parameters: sample size and smoothness. The smoothness parameter is the full-width-at-half-maximum (FWHM) of a Gaussian kernel which, when convolved with uncorrelated 1-D Gaussian data, would yield the same temporal smoothness as the average smoothness of the given dataset’s residuals. A robust procedure for estimating FWHM was introduced for n-dimensional data in (Kiebel et al., 1999) and has been validated for 1-D data in (Pataky, 2016).

Exactly as 0-D parametric inference assumes 0-D Gaussian randomness, RFT assumes 1-D Gaussian randomness. 0-D Gaussian randomness is parameterized by sample size, or more precisely: degrees of freedom, and 1-D Gaussian randomness is additionally parameterized by a smoothness parameter, the FWHM (Kiebel et al., 1999). However, since this assumption might be violated researchers are encouraged to check the normality of their data before conducting RFT analyses. One way is to use the D’Agostino-Person normality test (D’Agostino, Belanger & D’Agostino, 1990), which can be RFT-corrected (Pataky, 2012).

SPM’s applied use of RFT was developed in neuroimaging (Worsley et al., 1992; Friston et al., 2007) to control the false positive rate. SPM and RFT have recently spread to various fields such as Electrophysiology (Kiebel & Friston, 2004) and Biomechanics (Pataky, 2012) and have been validated for hypothesis testing for 1-D data (Pataky, 2016). Example uses of SPM in biomechanics include: dynamic comparisons of elite and recreational athletes (Mei et al., 2017), effects of chronic ankle instability on landing kinematics (De Ridder et al., 2015), and effects of shoe ageing on running dynamics in children (Herbaut et al., 2017).

A viable alternative to SPM’s false positive control during multiple hypothesis testing is to instead control the false discovery rate (FDR). The FDR represents the proportion of falsely rejected null hypotheses amongst all rejected null hypotheses when simultaneously testing multiple hypotheses (Benjamini & Hochberg, 1995). FDR inference uses the highest *p*-value satisfying the inequality *p* (i) ≤ i *α*/Q as a critical threshold, where *α* is the Type I error rate, usually 0.05, *i* is the index of the ordered *p*-values, and *Q* is the total number of tests. Thus, the FDR control procedure of (Benjamini & Hochberg, 1995) computes node-wise *p*-values and orders them to calculate the *p* threshold that ensures that the FDR is less than *α* over a large number of experiments. Usually inter-test independence is assumed (Benjamini & Hochberg, 1995) even if the assumption has little practical impact on the results (Benjamini, 2010; Chumbley et al., 2010).

Moreover, FDR procedures are generally less conservative than Type I error control across the Q tests and the adaptability of FDR thresholds to the data allow a balance of Type I and Type II errors (Benjamini & Hochberg, 1995; Storey & Tibshirani, 2003). FDR has been used as a thresholding technique for functional neuroimaging (Genovese, Lazar & Nichols, 2002; Chumbley & Friston, 2009; Schwartzman & Telschow, 2019) and has been described as a method that has the potential to eclipse competing multiple testing methods (Nichols & Hayasaka, 2003; Pike, 2011).

In biomechanics literature, FDR procedures have been used to correct multiple testing problems involving 0-D metrics (Matrangola et al., 2008; Horsak & Baca, 2013). However, to the best of our knowledge, no previous study has used FDR control to analyze 1-D data.

Although RFT inference is considered the most popular method to control family wise error rates in the neuroimaging literature (Lindquist & Mejia, 2015), the breakthrough FDR control paper (Benjamini & Hochberg, 1995) has led FDR control to become widely adopted in diverse fields such as: Neuroimaging (Genovese, Lazar & Nichols, 2002), bioinformatics (Reiner, Yekutieli & Benjamini, 2003), genomics (Storey & Tibshirani, 2003), metabolomics (Denery et al., 2010) and ecology (Pike, 2011). It has been argued that FDR control is more appealing than Type I error control because the former is more scientifically relevant than the latter (Genovese & Wasserman, 2002). That is, scientists are generally more interested in the proportion of nodes that are reported as false positives (FDR) than if there are any false positives (Type I error control). Thus, FDR has higher probability that the results declared significant correspond to an actual effect and not to chance.

The primary purpose of this study was to compare FDR and RFT thresholds in the analyses of 1-D data, and in particular to check whether these procedures could lead to qualitatively different interpretations of experimental datasets. To this end we reanalyzed a variety of publicly available datasets representing diverse experimental tasks (running, walking, cutting) and data modalities which span the breadth of biomechanical data including forces, kinematics and electrical muscle signals. These types of data have very different physical natures, are measured using very different equipment, and are generally processed in very different manners. For reporting purposes, we selected two datasets that most clearly illustrate the most relevant scientific implications of choosing between RFT and FDR. We also performed complementary numerical simulations, involving random (Gaussian) 1-D data, to explain the RFT and FDR results’ convergence and divergence that we observed in the experimental datasets.

## Materials & Methods

### Experimental datasets

Across a range of six public datasets in the spm1d software package (Pataky, 2012) from the Biomechanics literature (Neptune, Wright & van den Bogert, 1999; Pataky et al. 2008; Pataky et al. 2014; Besier et al. 2009; Caravaggi et al., 2010; Dorn, Schache & Pandy, 2012) we selected two datasets to report in the main manuscript (Table 1). The criteria for inclusion were: (1) one dataset exhibiting RFT-FDR convergence, (2) one dataset exhibiting RFT-FDR divergence, and (3) adherence of these two datasets to RFT’s normality assumption, so that the RFT results could reasonably be considered valid. Information regarding the remaining datasets, including the statistical analysis results, are available in Appendix E.

**Table 1:** Experimental datasets.

Dataset	Source	J	Q	Model	Task	Variable
A	Caravaggi et al., 2010	10	101	Paired *t*-test	Walking	Plantar arch deformation
B	Dorn, Schache & Pandy, 2012	7	100	Linear regression	Running/Sprinting	Ground reaction force

**Notes.**

J sample size Q number of time nodes

Dataset A (Caravaggi et al., 2010) consisted of plantar arch deformation data with the purpose of studying the relationship between the longitudinal arch and the passive stabilization of the plantar aponeurosis. Ground reaction force (GRF) data were collected from ten participants during walking at different speeds: slow, normal and fast walking. For each speed, participants performed ten trials over a wooden walkway with an integrated force plate to record stance-phase GRF. Here we consider only two of the study’s categorical speeds: “normal” and “fast”. Since each participant performed both speeds, the underlying experimental design was paired.

Dataset B (Dorn, Schache & Pandy, 2012) consisted of three-dimensional GRF data from seven participants during running and sprinting at four different speeds, slow running at 3.56 m/s, medium-paced running at 5.20 m/s, fast running 7.00 m/s and maximal sprinting at 9.49 m/s. Over a 110 m track, the participant accelerated to a steady state up to 60 m, held the steady state for 20 m and decelerated over the remaining 30 m. The data were collected during the steady state phase. Only two trials per speed were available, and only the mediolateral GRF component was analyzed. Speed effects were examined using linear regression analysis.

### Data analysis

The analyses in this paper were conducted in Python 3.6 (Van Rossum, 2018), using Anaconda 4.4.10 (Anaconda, Inc.) and the open source software packages: spm1d (Pataky, 2012) and power1d (Pataky, 2017). Software implementing FDR inferences for 1-D data (see text below) are available in this project’s repository: https://github.com/0todd0000/fdr1d.

For both datasets, first the test statistic (*t* value) was computed at each time node, yielding an “SPM{t}” as detailed elsewhere (Kiebel et al., 1999). Statistical inferences regarding this SPM{t} were then conducted by computing critical domain-wide thresholds. These thresholds were calculated using two different procedures: Type I error rate control using RFT and FDR control, the ratio of Type I errors to the number of significant tests. The two methods yielded two thresholds per dataset. The RFT thresholds were calculated based on estimated temporal smoothness (Kiebel et al., 1999) as detailed elsewhere (Friston et al., 2007; Pataky, 2016). The FDR thresholds were calculated according to (Benjamini & Hochberg, 1995) as detailed elsewhere (Genovese, Lazar & Nichols, 2002) and as described in this article’s Supplemental Information.

Both statistical methods (RFT and FDR) have corrected for Q comparisons, where Q is the number of time nodes in each dataset (Table 1). We also briefly considered 0-D (“Uncorrected”) and Bonferroni procedures in the context of 1-D smooth data to demonstrate the limitations of both in the analysis of 1-D measurements.

### Simulations

Numerical simulations involving smooth, random 1-D data were conducted with the goal of explaining the similarities and differences between the aforementioned RFT and FDR thresholds. Two sets of simulations were conducted: (i) qualitative experimental results replication, and (ii) RFT/FDR threshold divergence exploration.

### Replicating the experimental results

**Figure 1:**
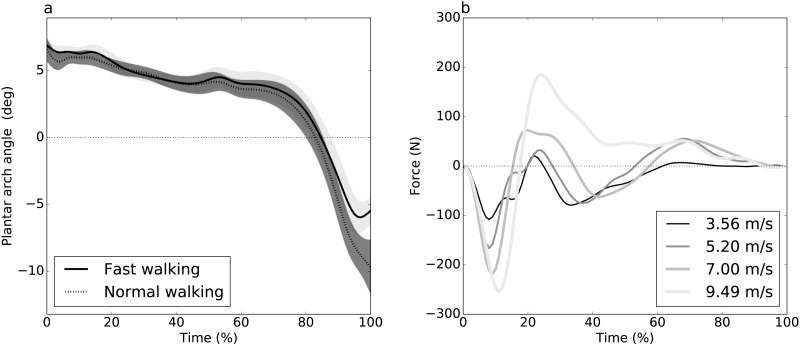
Experimental datasets. (A) Dataset A (Caravaggi et al., 2010): plantar arch angle in 10 participants during normal and fast walking (means and standard deviation clouds). (B) Dataset B (Dorn, Schache & Pandy, 2012): mediolateral ground reaction force during running/sprinting at four different speeds for one participant, means of two trials were shown.

**Figure 2:**
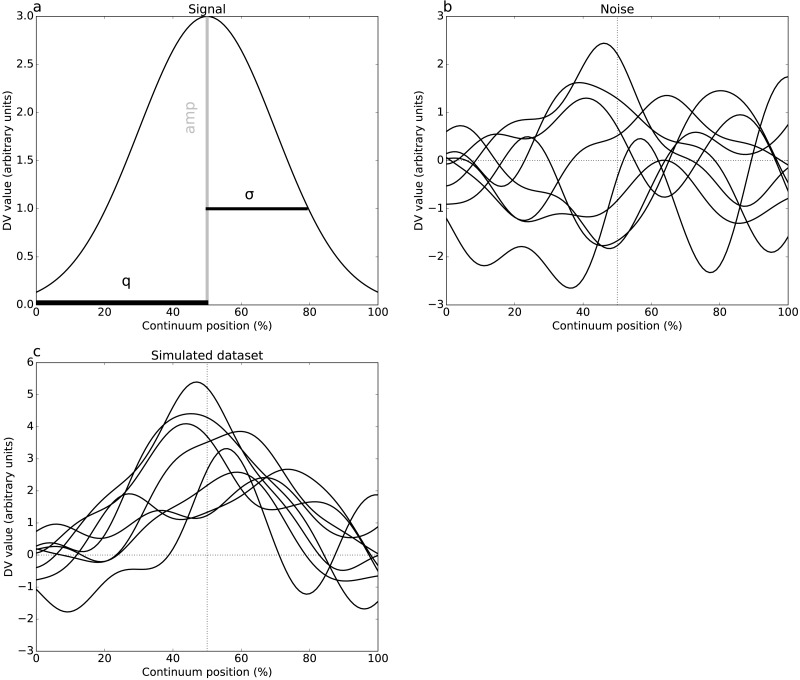
Simulated dataset example (DV = dependent variable). (A) Gaussian pulse, representing the true signal, and characterized by amplitude *amp* and standard deviation *σ*. (B) One-dimensional smooth Gaussian fields, representing the dataset residuals, and characterized by the smoothness parameter *FWHM* = 20% (full-width-at-half-maximum) (Kiebel et al., 1999). (C) Simulated dataset (signal + noise).

Two simulations were conducted, one per dataset (Fig. 1), involving both 1-D signal (Fig. 2A) and smooth 1-D noise (Fig. 2B). The signal was modeled as a Gaussian pulse, and parameterized by pulse center (q), pulse breadth (*σ*, standard deviation units) and amplitude (amp). These three parameters were manually adjusted so that, when added to random 1-D noise, the resulting simulated dataset (Fig. 2C) yielded statistical results that qualitatively replicated the experimental datasets’ results. (Table 2) lists the selected parameters which were estimated from experimental datasets as FWHM = 20.37 and 7.94 for Datasets A and B, respectively.

The 1-D noise was created using a previously validated 1-D random number generator (Pataky, 2016). This generator accepted three parameters: sample size (J), number of continuum nodes (Q), and smoothness estimate (FWHM). All noise parameters were selected to follow the experimental datasets (Tables 1–2). For these “replication” simulations, only two realizations of noise (and thus only two simulated datasets) were produced. These datasets were analyzed identically to the experimental datasets.

### Exploring threshold divergence

Both datasets without signal (Fig. 2B) and datasets with signal (Fig. 2C) were simulated. The aforementioned simulations were conducted using Monte Carlo simulations, involving manipulation of J and FWHM for datasets without signal and all aforementioned parameters except Q (i.e., J, q, *σ*, amp and FWHM) for the simulated datasets with signal. Sample size J was varied between 5 and 50, representing the small to moderate sample sizes typical in Biomechanics research (Knudson, 2017). Signal position q was varied between 0 and Q. Signal breadth *σ* was varied between 0 and 20. Signal amplitude was varied between 0 and 4; since the standard deviation of the noise is one, the latter corresponds to approximately four times the noise amplitude. The smoothness (FWHM) was varied between 10% and 30%, representing the set of previously reported smoothness values for biomechanical data (Pataky, Vanrenterghem & Robinson, 2015) that were found in this study to be sufficient to illustrate RFT/FDR divergence.

For each parameter combination, 10,000 simulation iterations were conducted, each involving a new noise realization. FDR thresholds were computed for each dataset, then averaged across the 10,000 iterations. For simulations without signal, RFT thresholds were also computed for every iteration. Convergence/divergence of the RFT and FDR thresholds were judged qualitatively, by plotting them as functions of the other simulation parameters. In interest of space we report only key simulation findings. Moreover, additional details and results, including code necessary to produce our results, are provided as Supplemental Information in this project’s public repository (https://github.com/0todd0000/fdr1d/).

**Table 2:** Qualitatively estimated simulation parameters which yielded similar results to the experimental datasets. Sample sizes were the same as in the original datasets. Noise smoothness (FWHM) was estimated from the experimental datasets following (Kiebel et al., 1999).

**Characteristic**	**Symbol**	(Simulated) **Dataset A**	(Simulated) **Dataset B**
Sample size	*J*	10	7
Signal center	*q*	101	17
Signal breadth	*σ*	3	19
Signal amplitude	*amp*	2.3	1.2
Noise smoothness	*FWHM*	20.37	7.94

## Results

### Experimental datasets results

#### Dataset A: plantar arch deformation during walking

**Figure 3:**
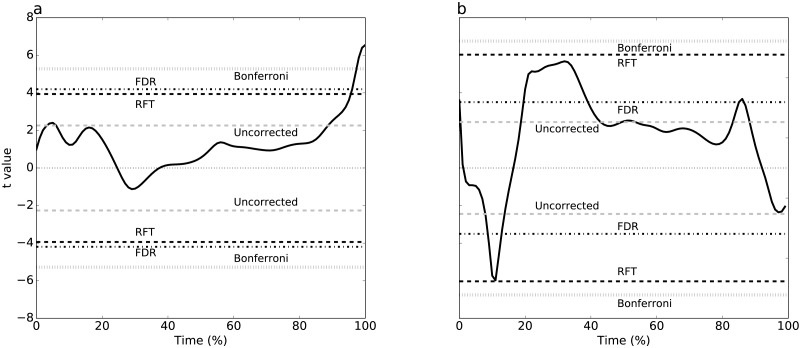
Experimental results, two tailed-tests. (A) Dataset A, (B) Dataset B. Four different thresholds are depicted: Bonferroni, false discovery rate (FDR), random field theory (RFT) and uncorrected. The null hypothesis is rejected if the *t* value traverses a threshold.

Plantar arch deformation in early-to mid-stance increased with walking speed (Fig. 1A). It reached its maximum deformation during late stance with fast walking exhibiting less deformation compared to normal walking (Fig. 1A). Statistical results suggested a rejection of the null hypothesis of no speed effects, with significant differences over 95% to 100% stance (Fig. 3A). The four critical thresholds were related as follows: Uncorrected <RFT<FDR<Bonferroni

#### Dataset B: GRF during sprinting

As running speed increased, the mediolateral GRF magnitude increased systematically, with relatively high magnitude during the first 50% of stance time (Fig. 1B). The four different analysis approaches (Uncorrected, FDR, RFT and Bonferroni) yielded contradictory results (Fig. 3B). While RFT and Bonferroni inferences failed to find significant correlation between speed and mediolateral GRF, uncorrected and FDR inferences yielded significance.

### Simulation results

#### Replicating the experimental results

**Figure 4:**
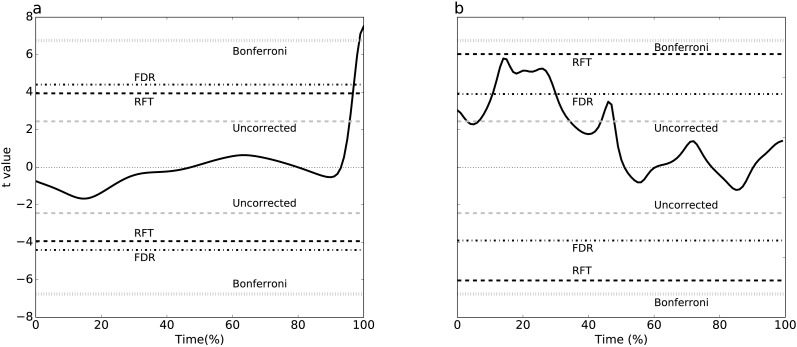
Simulation-based replication of the experimental results (Fig. 3). These results were created using the parameters listed in Table 2.

Simulating two experimental-like datasets (Fig. 4) yielded qualitatively similar results to the real experimental results (Fig. 3). In particular, a convergence of RFT and FDR thresholds was observed in both experimental and simulated analyses of the paired datasets (Figs. 3A–4A), and a divergence of thresholds was observed for the other dataset (Figs. 3B–4B). The signal parameters used to create these results (Table 2) suggest that RFT and FDR thresholds converge for small-amplitude, small-breadth signal, and that they diverge for large-amplitude, large-breadth signal. However, this result pertains to just two specific cases, so more systematic simulation results were used to verify these observations, as described below.

#### Exploring threshold divergence

**Figure 5:**
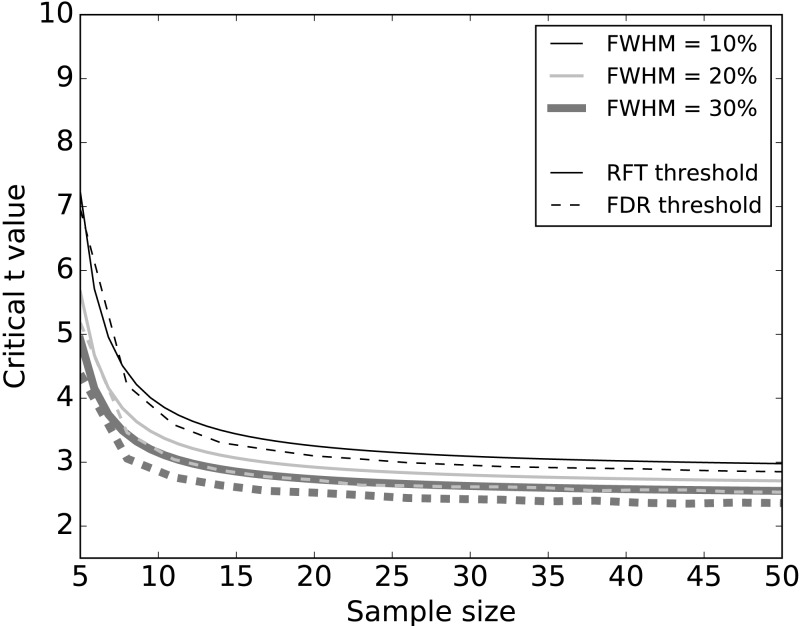
Simulation results: effects of 1–D smoothness (parameterized by the full-width at half maximum—FWHM) and sample size on critical thresholds. Results represent averages over 10,000 iterations.

Systematic simulations of datasets without signal (Fig. 5) found that FDR thresholds were slightly lower than RFT thresholds, irrespective of smoothness. Moreover, both RFT and FDR thresholds decreased with both smoothness (FWHM) and sample size. Since any given dataset embodies a single sample size and a single smoothness, these results suggest that FDR thresholds will be marginally smaller than RFT thresholds when there is no true signal.

**Figure 6:**
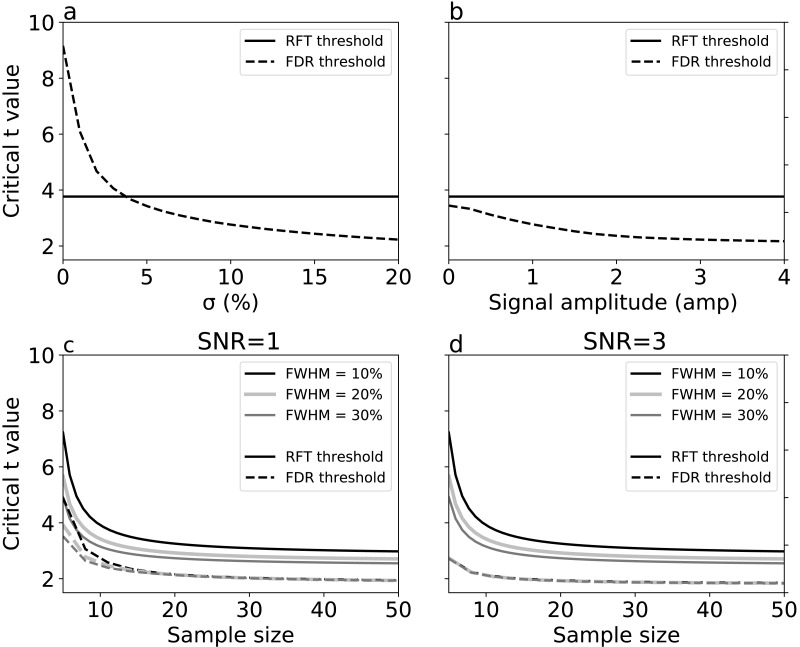
Simulation results: effects of (A) signal breadth *σ*, (B) signal amplitude, and (C, D) sample size and noise smoothness on critical thresholds. (C) and (D) depict signal-to-noise ratios (SNR) of 1 and 3, respectively.

For simulations with true signal, signal breadth (*σ*) (Fig. 6A) and signal amplitude (Fig. 6B) did not affect RFT thresholds, which are, by definition, dependent only on noise characteristics. FDR thresholds, in contrast, reduced with both signal breadth and signal amplitude. For low signal breadth values (*σ* <  5), the decrease of FDR thresholds was pronounced, from 9.15 to 3.42, and the FDR thresholds were greater than the RFT thresholds for very narrow signal breadths. The FDR thresholds decreased less markedly with signal amplitude (Fig. 6B), suggesting that signal breadth was a more important factor for determining the difference between RFT and FDR thresholds.

Last, we repeated the no-signal simulations (Fig. 5) in the face of signal, and found that, although the basic no-signal trends of decreased thresholds with sample size and smoothness were present (Figs. 6C–6D), these trends were dominated by the presence of signal, which pushed the FDR and RFT thresholds apart with respect to the no-signal case. These results suggest that the presence of a broad, true signal is the primary factor for causing RFT and FDR threshold divergence.

## Discussion

We considered RFT and FDR as alternative multiple testing solutions in the context of both arbitrary, simulated 1-D data, and in the context of open-source experimental data from Biomechanics. For context, uncorrected and Bonferroni procedures were also considered.

Our simulation results show that FDR and RFT thresholds converge in the absence of signal, and diverge in the presence of signal, in agreement with previous reports from the 3-D Neuroimaging literature (Nichols & Hayasaka, 2003; Schwartzman & Telschow, 2019). To our knowledge, no previous study has explicitly compared FDR and RFT in the context of smooth 1-D data. Our simulations showed that the extent to which RFT and FDR inferences diverged can be quite large, especially when the true signal is large and/or spans a large portion of the 1-D domain. This finding is non-trivial because signal-dependent divergence of competing analysis techniques implies that non-trivial interpretation differences may result from technique choice, and thus that investigators could conceivably choose, in an *ad hoc* manner, the technique that more closely supports a particular perspective.

For example, for Dataset A (Fig. 3A), 0-D and 1-D procedures both implied that plantar arch deformation late stance was significantly higher for normal walking group compared to fast walking. This interpretation is in accordance with the original paper’s findings (Caravaggi et al., 2010). In other words, while the two thresholds were not numerically identical, their differences were small with respect to the test statistic magnitude, implying an effectively identical interpretation.

Dataset B exhibited RFT and FDR threshold divergence (Fig. 3B) which yielded opposite conclusions. Since the test statistic failed to cross RFT and Bonferroni thresholds, there was a lack of evidence that the mediolateral forces are affected by running speed and, by consequence, the null hypothesis would not be rejected. However, FDR and uncorrected thresholds were considerably lower for this dataset and the test statistic crossed these thresholds at three intervals: 2% to 18%, 20% to 40% and 82% to 85% stance time. This suggests sufficient evidence to reject the null hypothesis that the mediolateral forces are unaffected by speed. This interpretation promotes a 3-D perspective on running, in which dynamics in the task-relevant direction (i.e., anterior direction) are inseparable from dynamics in other directions. This interpretation difference may seem subtle, but it is analogous to a view of human running as robotic, in which single-actuators control single-direction movements, as opposed to a view of human running as decidedly more complex, involving complex actuators driving complex 3-D structures.

We cannot compare our Dataset B results to those from the main paper (Dorn, Schache & Pandy, 2012) because the authors of that study did not explicitly analyze mediolateral forces. Nevertheless, we can compare these divergent interpretations with other published studies. For example, it has been shown that mediolateral forces can increase approximately two-fold in walking and up to four-fold in running (Nilsson & Thorstensson, 1989). In contrast, it has also been reported that mediolateral force variability is considerably larger than speed-related changes (Munro, Miller & Fuglevand, 1987). Contradictory findings like these are generally problematic. This study’s results show that convergence and divergence of statistical results can occur even in single experimental datasets when different analysis techniques are used, thereby leading to contradictory scientific conclusions (Fig. 3B). Consequently, it is possible to choose amongst competing, valid analysis techniques, and to thereby promote a particular scientific viewpoint in an *ad hoc* manner. To avoid this problem, it is important to choose the statistical approach prior to conducting statistical analyses depending on the experimental design and the goals of the study to avoid the “significance chase” which has been considered one of the main reasons behind the confidence crisis in science (Ioannidis, 2007). Alternatively, if the study is exploratory, it may be prudent to choose a variety of techniques in an *a priori* manner and to report where those techniques’ results differ.

The 0-D and 1-D procedures considered in this study make different assumptions. Most simply, 0-D procedures assume 0-D randomness while 1-D procedures assume 1-D randomness. Since these statistical procedures yield different probabilities by definition, both 0-D and 1-D procedures cannot be considered valid for a given study. If one formulates a hypothesis regarding 0-D metrics in an *a priori* manner, then 0-D procedures are appropriate. However, if one conducts the experiment without *a priori* formulation of such hypotheses, then implicitly null hypotheses like “no effect” pertain to the entire 1-D domain, in which case 1-D procedures are appropriate.

Bonferroni thresholds assume 0-D randomness and also that the Q tests (across the 1-D domain) are independent. However, since the 1-D data are temporally smooth, and since the Bonferroni procedure fails to consider this smoothness, this procedure is generally overly conservative (c.f. Duhamel et al., 2004).

The primary advantage of RFT inference is that it is the simplest parametric form of hypothesis testing that appropriately handles smoothness to accurately control *α* in the analysis of 1-D data. It is simple because, for 1-D data, its parametric probabilities can be calculated based on only two parameters: sample size and field smoothness (FWHM). FWHM estimates can be calculated in a robust and unbiased manner (Kiebel et al., 1999) and have been shown to be robust to non-uniform smoothness for 1-D data (Pataky et al., 2019).

We conducted simulations to systematically examine the effects of FWHM in different 1-D SNR environments (Figs. 5 and 6). As we increased the smoothness parameter, RFT thresholds reduced and FDR thresholds -in the absence of signal- remained slightly lower than RFT thresholds, irrespective of smoothness level. However, in the presence of high SNR (Fig. 6D), FDR thresholds were lower than RFT thresholds, and the effects of the different smoothness levels on FDR inference diminished in accordance with previous results (Chumbley & Friston, 2009; Chumbley et al., 2010).

Sample size is an important consideration in an experimental design, representing a balance between statistical power and experimental resource conservation. Our numerical simulations showed a common pattern irrespective of the noise and SNR (Figs. 5 and 6). For sample sizes greater than 15, the effects of increasing sample size were negligible on the RFT and FDR thresholds. RFT thresholds for sample sizes lower than 15 were higher than FDR thresholds and were less affected by the presence of true signal. In contrast, FDR thresholds lowered as the SNR increased, irrespective of the sample size in the range of 5 to 50, which represents the small to moderate sample sizes typically found in the Biomechanics literature (Knudson, 2017).

While RFT offers whole-field control of *α* across the 1-D domain with two parameters (FWHM and sample size), FDR uses just one parameter (sample size) to achieve similar 1-D domain control. FDR does not directly control *α*, but instead controls the proportion of the falsely rejected null hypotheses, a quantity that has been argued to embody greater scientific relevance than multiple testing correction methods such as RFT (Genovese & Wasserman, 2002). Moreover, the FDR procedure is more powerful as it adapts to the signal features, embodying greater sensitivity for detecting true signal (Komiyama et al., 2017). Thus, FDR’s primary advantages are: simplicity, scientific relevance, and signal adaptability.

A practical advantage common to RFT and FDR is computational efficiency, because both compute probabilities parametrically. An investigation of parametric and nonparametric FDR estimation has shown that parametric FDR estimates are more accurate and efficient than nonparametric estimates (Wu, Guan & Zhao 2006). Analytical, parametric probability calculations execute very quickly compared to similar nonparametric permutation-based calculations which have been proposed for analyzing 1-D and n-D data (Nichols & Hayasaka, 2003; Pataky, Vanrenterghem & Robinson, 2015). Parametric procedures nevertheless rely on more assumptions than nonparametric methods, so before applying RFT and FDR analyses to their data, researchers should generally check if their data adhere to those assumptions including normality (Pataky, Vanrenterghem & Robinson, 2016).

Since FDR inference adapts to changes in the signal, it is advisable to maximize the SNR (i.e., minimize noise) to ensure optimal FDR power. This can be achieved through procedures like digital filtering (Winter, Sidwall & Hobson, 1974; Challis, 1999) which remove artifacts, high frequency noise, and other signal contaminants. Nevertheless, when noise is confined to a small portion of the 1-D measurement, it is expected to have little impact on FDR results (Genovese, Lazar & Nichols, 2002).

The nature of biomechanical data and technological advancements allow researchers to collect hundreds-to-thousands of dependent variables. RFT and FDR thresholds are generally both valid procedures for multiple testing across these variables. However, FDR generally maintains greater statistical power than RFT, thereby maximizing discoveries of true signal. Thus, when aiming to maximize discoveries during multiple testing problems, FDR may be a better choice.

Moreover, FDR control since its introduction (Benjamini & Hochberg, 1995) has shifted the scientific community’s thinking regarding multiple testing problems. Its intuitive concept and its adaptive behavior span a large range of multiple testing problems, and this has appealed to scientists from a variety of fields. Various FDR developments have been shown to produce even better results in certain situations (Storey, 2002; Storey & Tibshirani, 2003; Blanchard & Roquain, 2009; Gavrilov, Benjamini & Sarkar, 2009). However, some proposed FDR changes have been shown to not guarantee the desired FDR control (Benjamini, 2010; White, Van der Ende & Nichols, 2019) as the original procedure (Benjamini & Hochberg, 1995). We therefore limited our analyses to the originally proposed FDR correction.

## Conclusions

This study considered FDR control as an alternative to false positive rate control (i.e., RFT-based control) in the context of 1-D continuum data analysis, using examples from Biomechanics. FDR and RFT were compared using both open experimental datasets and numerical, Monte Carlo simulations.

Results showed that FDR is sensitive to 1-D signal changes, and that the procedure is less restrictive and more sensitive than other multiple testing methods. FDR would be useful for studies that aim to maximize discovery, and RFT would likely be more useful for hypothesis-driven studies, in which sample sizes, signal characteristics, and variance characteristics can be considered simultaneously in order to balance false positive and false negative control.

## Acknowledgements

We would like to thank the editor and the anonymous reviewers who have helped us reshape our manuscript to reach broader readership, with their insightful comments and suggestions. Furthermore, we thank PeerJ staff for their efficiency in making the review process a smooth one.

##  Supplemental Information

10.7717/peerj.8189/supp-1 Supplemental Information 1 Appendix E: Experimental datasets This appendix contains RFT and FDR results (Fig. E1) from six experimental datasets and a total of eight different analyses (Table E1) that were conducted but were not included in the main manuscript. The datasets represent a variety of biomechanical modalities, experimental designs and tasks.

## Additional Information and Declarations

### Data Availability

The following information was supplied regarding data availability: The data is available under “False discovery rate (FDR) inferences for one-dimensional (1-D) data”: https://github.com/0todd0000/fdr1d.
