# Supplementary material for: A comparison of random-field-theory and false-discovery-rate inference results in the analysis of registered one-dimensional biomechanical datasets
Source: PeerJ. 2019 Dec 10;7:e8189. doi: 10.7717/peerj.8189 (PMC6910120; doi:10.7717/peerj.8189)
Supplement: Supplemental Information 1 — This appendix contains RFT and FDR results (Fig. E1) from six experimental datasets and a total of eight different analyses (Table E1) that were conducted but were not included in the main manuscript. The datasets represent a variety of biomechanical modalities, experimental designs and tasks. [file peerj-07-8189-s001.pdf]

# Appendix E: Experimental datasets

This appendix contains RFT and FDR results (Fig.E1) from six experimental datasets and a total of eight different analyses (Table E1) that were conducted but were not included in the main manuscript. The datasets represent a variety of biomechanical modalities, experimental designs and tasks.

**Table E1**. Experimental datasets and analyses. J and Q are the sample size and number of time nodes, respectively. GRF = ground reaction force. EMG = electromyography.

| Dataset | Name | J | Q | Model | Task | Variables |
|---|---|---|---|---|---|---|
| A | Caravaggi et al., 2010 | 10 | 101 | Paired t-test | Walking | Plantar arch deformation |
| B | Dorn, Schache & Pandy, 2012 | 7 | 100 | Linear regression | Running/ sprinting | GRF |
| C | Pataky et al., 2008 | 59 | 101 | Linear regression | Walking | GRF |
| D | Neptune, Wright & Van Den Bogert, 1999 | 15 | 101 | Two sample t-test | Cutting movement | Kinematics, EMG |
| E | Pataky et al., 2014 | 10 | 101 | Paired t-test | Walking | Center of pressure |
| F | Caravaggi et al., 2010 | 19 | 101 | Two sample t-test | Walking | Plantar arch deformation |
| G | Pataky et al., 2008 | 20 | 101 | One sample t-test | Walking | GRF |
| H | Besier et al., 2009 | 40 | 100 | Two sample t-test | Walking, running | GRF, muscle forces |

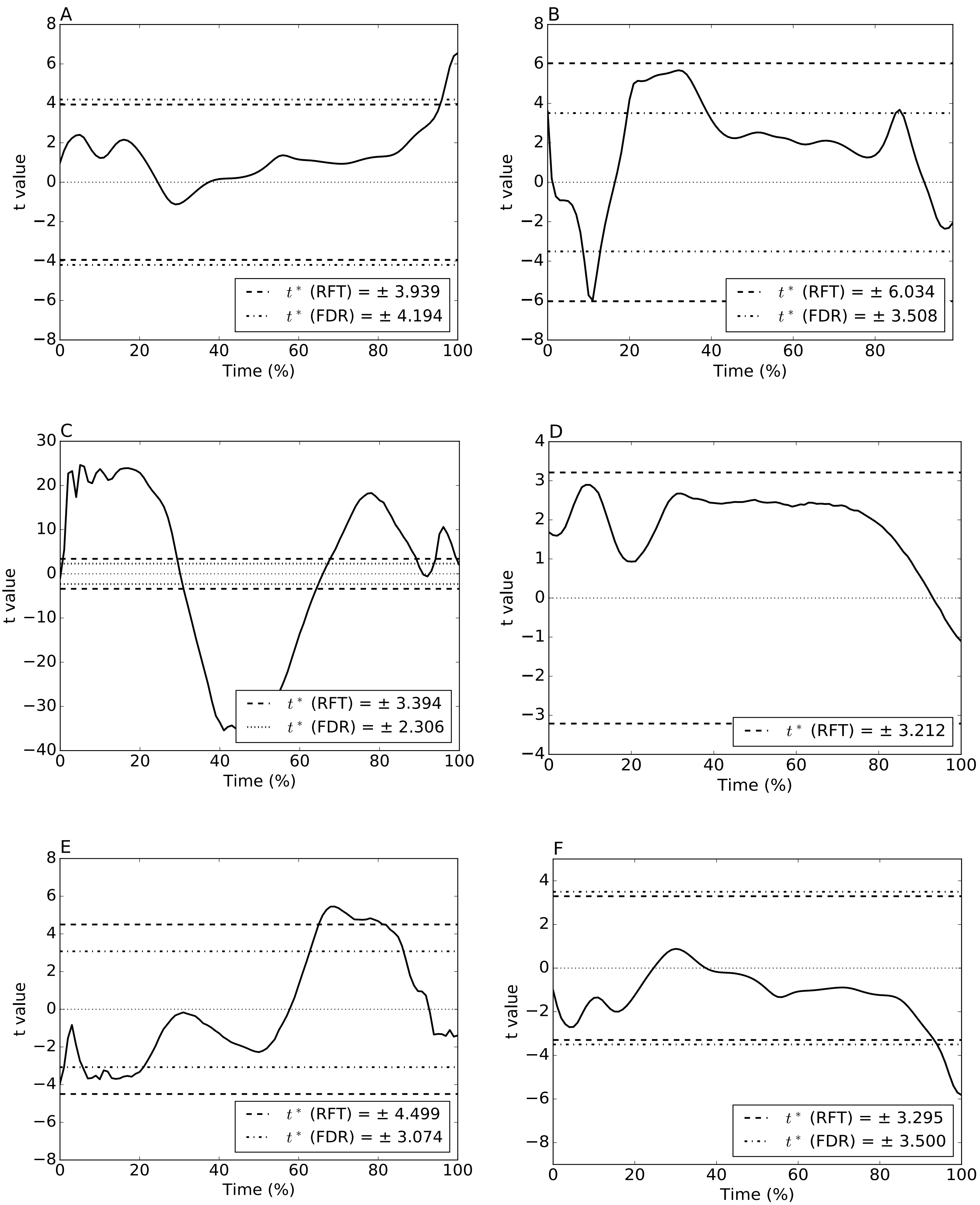
A
B
C
D
E
F
t value
Time (%)
$t^*$ (RFT) = ± 3.939
$t^*$ (FDR) = ± 4.194
$t^*$ (RFT) = ± 6.034
$t^*$ (FDR) = ± 3.508
$t^*$ (RFT) = ± 3.394
$t^*$ (FDR) = ± 2.306
$t^*$ (RFT) = ± 3.212
$t^*$ (RFT) = ± 4.499
$t^*$ (FDR) = ± 3.074
$t^*$ (RFT) = ± 3.295
$t^*$ (FDR) = ± 3.500

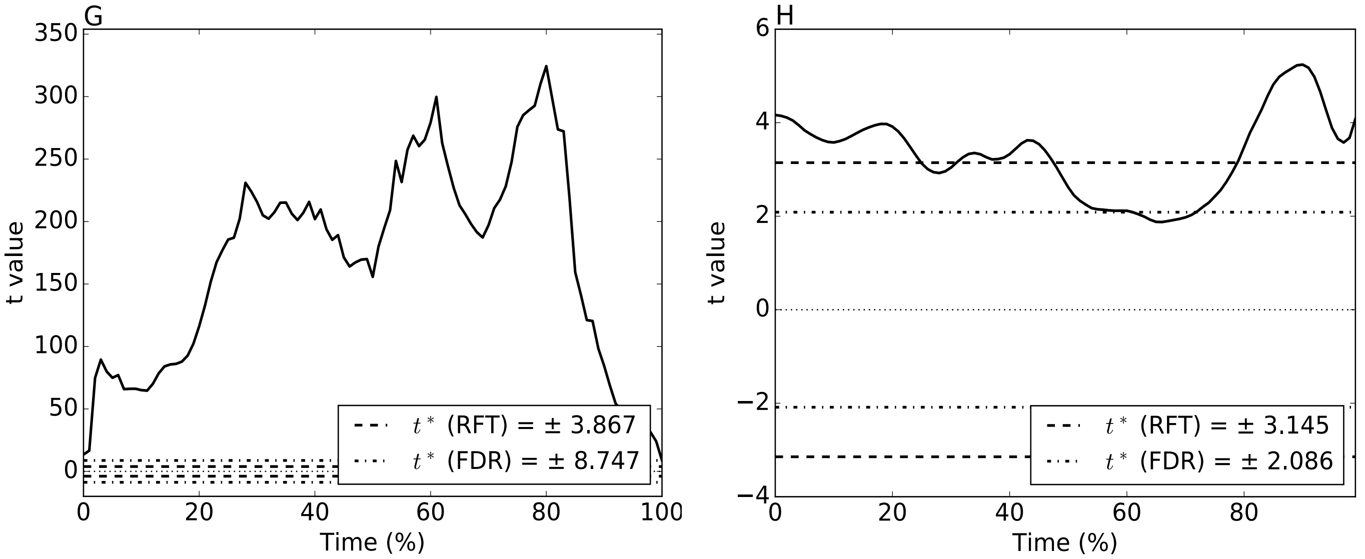


**Figure E1.** Results from six datasets depicting two thresholds: false discovery rate (FDR) and random field theory (RFT). The null hypothesis is rejected if the t value traverses a threshold.

**References:**


Besier TF, Fredericson M, Gold GE, Beaupré GS, Delp SL. 2009. Knee muscle forces during walking and running in patellofemoral pain patients and pain-free controls. *Journal of Biomechanics* 42:898–905. DOI: 10.1016/j.jbiomech.2009.01.032.

Caravaggi P, Pataky T, Günther M, Savage R, Crompton R. 2010. Dynamics of longitudinal arch support in relation to walking speed: Contribution of the plantar aponeurosis. *Journal of Anatomy* 217:254–261. DOI: 10.1111/j.1469-7580.2010.01261.x.

Dorn TW, Schache AG, Pandy MG. 2012. Muscular strategy shift in human running: dependence of running speed on hip and ankle muscle performance. *Journal of Experimental Biology* 215:1944–1956. DOI: 10.1242/jeb.064527.

Neptune RR, Wright IC, Van Den Bogert AJ. 1999. Muscle coordination and function during cutting movements. *Medicine and Science in Sports and Exercise* 31:294–302. DOI: 10.1097/00005768-199902000-00014.

Pataky TC, Caravaggi P, Savage R, Parker D, Goulermas JY, Sellers WI, Crompton RH. 2008. New insights into the plantar pressure correlates of walking speed using pedobarographic statistical parametric mapping (pSPM). *Journal of Biomechanics* 41:1987–1994. DOI: 10.1016/j.jbiomech.2008.03.034.

Pataky TC, Robinson MA, Vanrenterghem J, Savage R, Bates KT, Crompton RH. 2014. Vector field statistics for objective center-of-pressure trajectory analysis during gait, with evidence of scalar sensitivity to small coordinate system rotations. *Gait and Posture* 40:255–258. DOI: 10.1016/j.gaitpost.2014.01.023.
